# Thymic Dendritic Cells Revisited

**DOI:** 10.1111/imr.70076

**Published:** 2025-11-18

**Authors:** Matouš Vobořil, Shuya Xuan, Kristin A. Hogquist

**Affiliations:** ^1^ Department of lab Medicine and Pathology, Center for Immunology University of Minnesota Medical School Minneapolis Minnesota USA; ^2^ Department of Cell Biology, Faculty of Science Charles University Prague Czech Republic

**Keywords:** cell lineages and subsets, T cells, thymus, tissues, tolerance

## Abstract

Central tolerance in the thymus ensures that the developing T cell repertoire is safe yet effective against infections. This process relies greatly on antigen presentation by both stromal and hematopoietic antigen‐presenting cells (APCs), with dendritic cells (DCs) playing a particularly critical role. Thymic DCs acquire a broad spectrum of self‐antigens, including tissue‐restricted antigens (TRAs), inflammation‐associated antigens (ISAs), and peripheral antigens imported via circulation or immigrating DCs. These diverse inputs allow DCs to mediate clonal deletion, regulatory T cell (Treg) induction, and other agonist selection outcomes. In this review, we revisit thymic DCs, outlining their ontogeny, transcriptional control, and functional specialization. We compare thymic DC1 and DC2 subsets with their peripheral counterparts, highlighting their distinct localizations, maturation cues, and division of labor: DC1 excel in cross‐presentation and Treg generation, while DC2 preferentially drive clonal deletion. We also highlight the heterogeneity of DC2s, which consist of four distinct subsets based on their transcriptional and phenotypic programs. We further examine plasmacytoid DCs, transitional DCs, monocytes, and macrophages, which contribute to tolerance through apoptotic cell clearance, antigen transfer, and lineage diversion of thymocytes. Finally, we discuss the role of homeostatic maturation, sterile inflammatory cues, and thymic immigration in shaping APC diversity. Together, these insights underscore the heterogeneity of thymic APCs, the complexity of thymic DC biology, and its vital importance in enforcing immune self‐tolerance.

## 1 Introduction to Antigen Presentation in the Thymus

**FIGURE 1:**
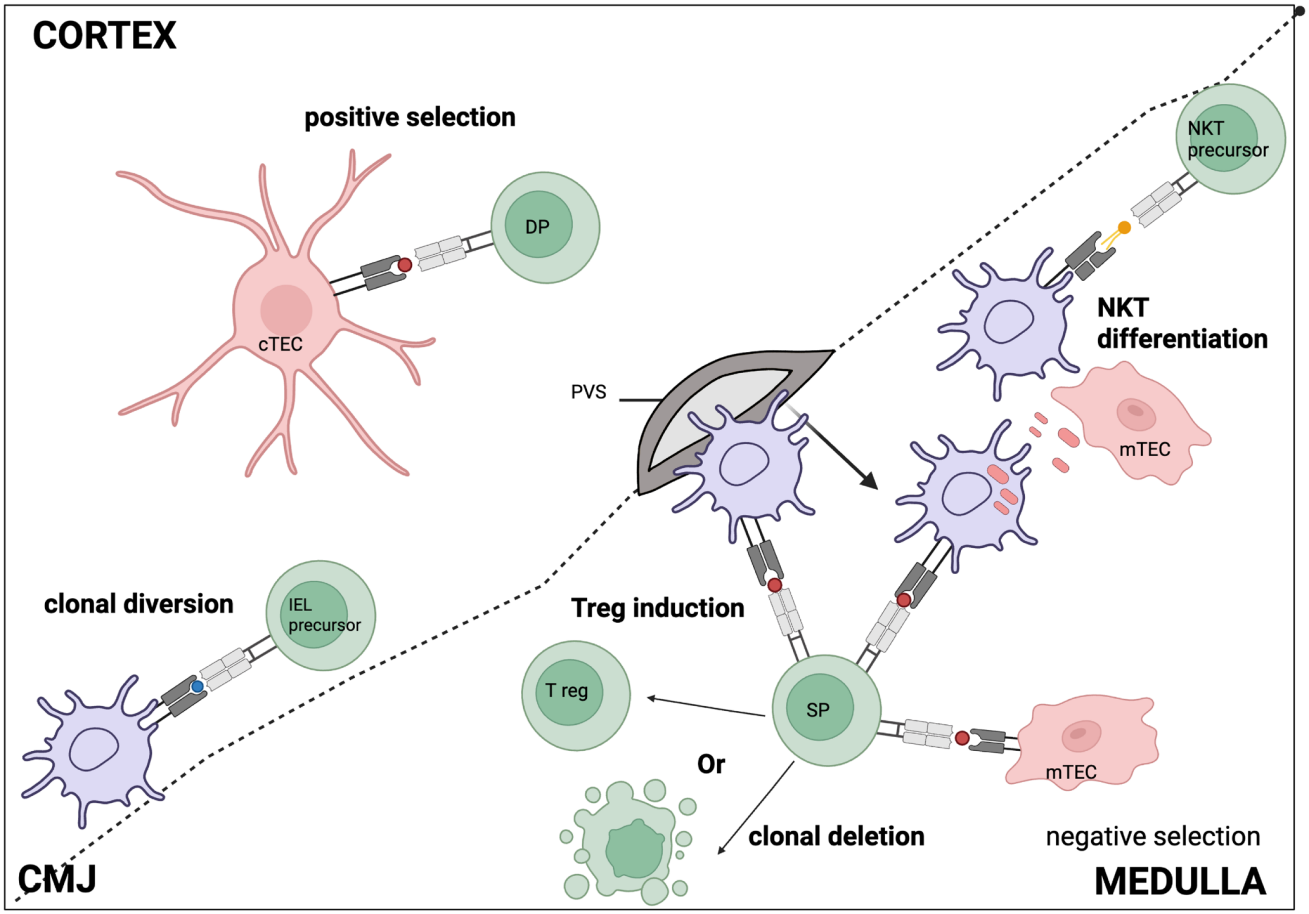
Thymic APCs orchestrate distinct stages of T cell selection. Developing double‐positive (DP) thymocytes in the cortex undergo positive selection through interactions with peptide–MHC (pMHC) complexes presented by cortical thymic epithelial cells (cTECs). Thymocytes with functional TCRs migrate to the medulla as single‐positive (SP) cells, where medullary thymic epithelial cells (mTECs) and hematopoietic antigen‐presenting cells (APCs) mediate negative selection. Self‐reactive SP thymocytes either undergo clonal deletion or are induced into the regulatory T cell (Treg) lineage. Cooperative antigen transfer (CAT) from mTECs to hematopoietic APCs ensures broad self‐antigen availability for tolerance induction. In addition, specialized interactions at the corticomedullary junction (CMJ) contribute to the development of invariant NKT (iNKT) and intraepithelial lymphocytes (IEL), from lipid specific (yellow) and self‐reactive (blue) precursors, respectively.

The adaptive immune system provides a powerful tool to fight against a wide range of pathogens through the expression of a highly diverse T cell receptor (TCR) repertoire. This diversity arises from random gene rearrangement, enabling T cells to recognize virtually any antigen, including self‐antigens. This could be potentially dangerous if self‐reactive T cells are not properly regulated and could lead to autoimmunity. To prevent the development of pathological autoimmune reactions, self‐reactive T cells are physically or functionally eliminated in the thymus through a process known as central tolerance [1]. Within the thymus, self‐reactive T cells can be either eliminated through clonal deletion or redirected into different T cell lineages such as regulatory T cells (Tregs), invariant natural killer T cells (iNKT cells), intraepithelial lymphocytes (IEL), or mucosal‐associated invariant T (MAIT) cells through a mechanism collectively referred to as agonist selection [2, 3] (Figure 1). These processes ensure that the conventional T cell repertoire remains both effective and self‐tolerant.

Central tolerance is orchestrated through the presentation of self‐antigens by peptide‐ MHC complexes (p:MHC) on various antigen‐presenting cells (APCs), which interact with developing thymocytes. In addition to the self‐antigens expressed by thymic APC, the T cell repertoire needs to be tolerant to self‐antigens expressed by APC in peripheral tissues as well, which may be considerably different. The presentation of peripheral self‐antigens within the thymus occurs through multiple mechanisms. One is the ectopic expression of thousands of tissue‐specific antigens (TSAs) by medullary thymic epithelial cells (mTEC). This process is partially regulated by a specialized transcriptional regulator, Autoimmune Regulator (AIRE) [4]. In addition, mature mTECs can initiate molecular programs that mimic the differentiation of peripheral epithelial cell types. As a result, the thymus contains cells that resemble tuft cells, hepatocytes, neuroendocrine cells, microfold cells, and others. These cells, collectively known as ‘mimetic cells’, functionally expand the spectrum of TSAs presented in the thymus, for the purpose of T cell selection [5, 6].

Recent studies have shown that mTECs, along with other thymus‐resident cell types such as iNKT, γδ T cells, and eosinophils [7, 8], produce low‐level inflammatory signals in the thymus in the absence of infection or injury. These tonic inflammatory signals expand the repertoire of self‐antigens presented in the thymus to include inflammation‐associated self‐antigens (ISAs) [9, 10]. Interestingly, this sterile inflammatory environment not only broadens the spectrum of self‐antigens available for central tolerance, but also dramatically reshapes the thymic microenvironment and regulates the development of multiple APCs subpopuations [11, 12]. It induces “inflammatory states” in various thymic APCs, leading to notable heterogeneity, particularly among hematopoietic‐derived APCs (hAPCs), such as monocyte‐derived cells and dendritic cells.

**FIGURE 2:**
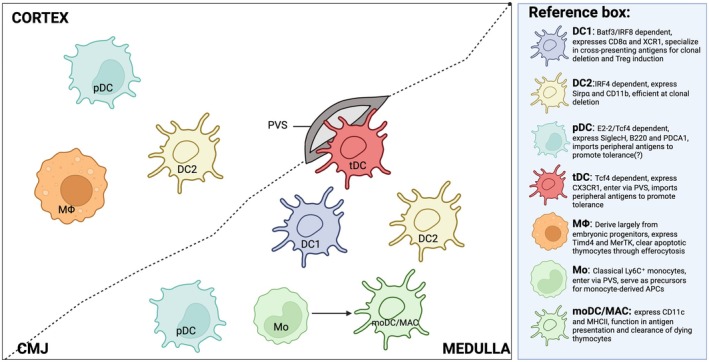
Heterogeneity and localization of thymic hematopoietic cell‐derived antigen‐presenting cells. Thymic myeloid APCs comprise multiple specialized subsets that collectively shape central tolerance. Dendritic cell 1 (DC1) is enriched in the medulla and corticomedullary junction (CMJ), where it excels at cross‐presenting antigens to mediate clonal deletion. Dendritic cell 2 (DC2) resides in both the cortex and medulla and promotes CD4^+^ T cell selection and Treg induction. Plasmacytoid DCs (pDCs) localize to the cortex and medulla, contributing to tolerance via peripheral antigen import. Transitional DCs (tDCs), enter the thymus from peripheral tissues at the perivascular space (PVS). Thymic macrophages (MФ) participate in efferocytosis and are abundant in the cortex. Monocytes (Mo) and monocyte‐derived DCs/macrophages (moDCs/MACs) reside in the medulla and contribute to antigen presentation and T reg induction.

The thymus hosts a diverse spectrum of hAPCs, including B cells, macrophages and DCs (Figure 2). Their importance in thymic selection was first demonstrated in mice lacking MHCII expression specifically in the hematopoietic lineage, while maintaining MHCII expression on stromal cells. This configuration allowed for the intact positive selection of T cells but resulted in impaired negative selection and increased susceptibility to autoimmunity [13, 14].

The thymus also contains diverse subpopulations of DCs [15], which predominantly reside in the medullary region, where they contribute to the establishment of central tolerance by either promoting clonal deletion of self‐reactive thymocytes or inducing Tregs generation [16, 17] (Figure 1) Furthermore, thymic DCs have been shown to efficiently acquire and cross‐present self‐antigens derived from mTECs to developing T cells [18, 19]. Taken together, these findings suggest that the extent of central tolerance depends on the diversity of self‐antigens encountered by thymocytes, with various thymic APCs, and thymic DCs in particular, playing a critical role in this process.

In this review, we highlight the complexity of thymic DCs, focusing on their ontogeny, development, intrathymic distribution, and roles in thymic antigen presentation and T cell tolerance. We also discuss the division of labor between thymic DC subsets in T cell selection, as well as the factors influencing their intrathymic maturation. Finally, we explore recently identified populations of thymic monocyte‐derived cells and transitional DCs (tDCs) [20], outlining their roles in thymic immigration and the induction of immune tolerance.

## 2 Thymic Dendritic Cells

Thymic APCs are broadly classified as being of either stromal origin (i.e., TECs) or of bone marrow origin (BM), which arise from hematopoietic stem cells (i.e., hAPCs). The importance of hAPCs in central tolerance has been supported by decades of research, anchored by two key observations. First, deletion or silencing of MHC II expression in BM‐derived cells increases the total number of CD4^+^ and CD8^+^ thymocytes, implicating their role in T cell clonal deletion [14, 21, 22, 23]. Second, studies using TCR transgenic models have shown that both mTECs and hAPCs are capable of inducing Treg selection upon presentation of cognate antigens [22, 23, 24, 25, 26, 27]. Among thymic hAPCs, DCs are the best characterized and most extensively studied.

DCs are sentinel immune cells specialized in sampling, capturing, and presenting antigens to naïve T cells to initiate immune responses, thereby bridging innate and adaptive immunity. Upon pathogen encounter, DCs become activated through pattern recognition receptor (PRR) signaling, which triggers widespread transcriptional and functional reprogramming, enabling them to migrate into lymphoid tissues and mount an appropriate T cell response [28, 29, 30]. Activated DCs (aDCs) upregulate MHC class II molecules along with co‐stimulatory molecules, cytokines, chemokine receptors and adhesion molecules, thereby establishing stable interactions with T cells and undergoing further “licensing” to become even more effective APCs [29, 31, 32].

**FIGURE 3:**
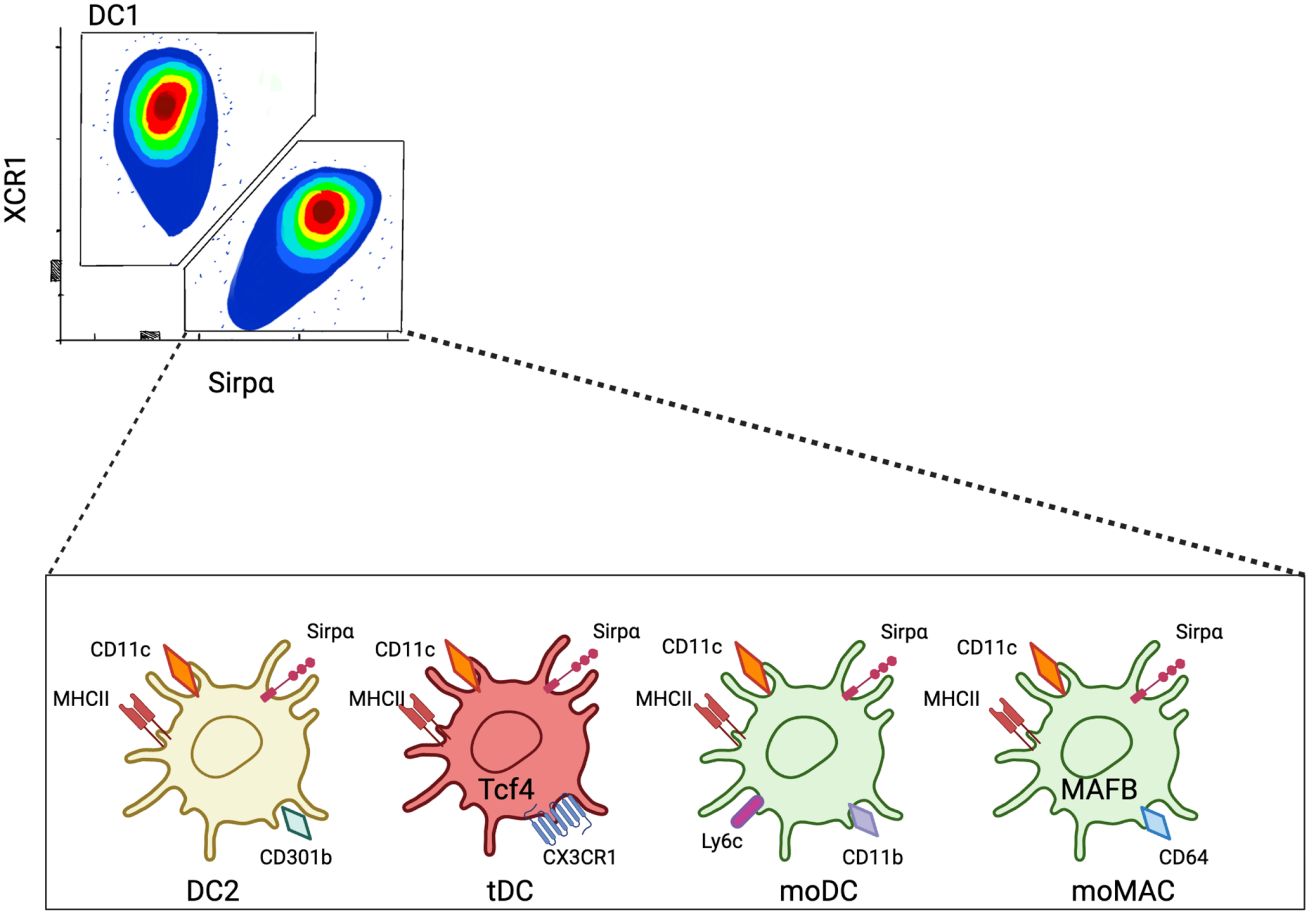
Subdivision of thymic DC2s based on transcriptional and phenotypic programs. Flow cytometry distinguishes DC1 and DC2 by canonical markers, with DC2 further divided into transcriptionally and phenotypically distinct subsets. DC2s are defined by CD11c, MHCII, Sirpα, and CD301b expression, features that support efficient presentation of self‐antigens to CD4^+^ thymocytes and promote Treg induction. Transitional DCs (tDCs), regulated by TCF4, express CD11c, MHCII, and CX3CR1, representing an intermediate state that may facilitate peripheral antigen import. Monocyte‐derived DCs (moDCs) display CD11c, MHCII, CD11b, and Ly6c, linking them to inflammatory recruitment and apoptotic thymocyte clearance. Monocyte‐derived macrophages (moMACs), controlled by MAFB, express CD11c and MHCII but are specialized for efferocytosis rather than priming.

In the thymus, DCs constitute only approximately 0.5% of total cellularity and are defined by the CD11c expression and varying levels of MHC class II [33]. Thymic DCs include plasmacytoid DCs (pDCs) and conventional/classical DCs (cDCs) [34]. Based on the surface marker expression, thymic cDCs are further divided into SIRPα^−^CD8α^+^ XCR1^+^ and SIRPα^+^CD8α^−^ XCR1^−^ subsets, resembling the classical peripheral DC1–DC2 dichotomy [34, 35]. Thus, thymic CD11c^+^MHCII^+^XCR1^+^ are referred to as DC1, while CD11c^+^MHCII^+^SIRPα^+^ are referred to as DC2 [35]. However, recent advances using single‐cell transcriptomics and lineage‐tracing mouse models have revealed that the CD11c^+^MHCII^+^SIRPα^+^ compartment is far more heterogeneous, containing not only DC2 but also monocyte‐derived cells and macrophages [20, 36] (Figure 3). Developmentally, cDCs, monocytes, and monocyte‐derived cells originate from a common myeloid progenitor (CMP), which differentiates into a monocyte/DC progenitor (MDP). The MDP subsequently gives rise to two lineages: the common DC progenitor (CDP), which develops into pre‐DC1 and pre‐DC2, and the common monocyte progenitors (cMoPs), which generate monocytes [29, 37]. In contrast, pDCs arise primarily from lymphoid progenitors [38]. The following sections will detail the ontogeny, development, characteristics and functions of individual thymic DC subsets.

### 2.1 Plasmacytoid Dendritic Cells (pDC)

As outlined above, DCs are divided into functionally specialized populations, including DC1, DC2 and pDCs [34]. pDCs represent a phenotypically and functionally distinct population of DCs, defined as CD11c^lo^CD11b^−^ SiglecH^+^B220^+^PDCA‐1^+^ cells [39] (Figure 2). Unlike cDCs, which are specialized for antigen presentation, naïve T cell priming, and T cell polarization, pDCs possess the ability to produce large amounts of type I interferons and pro‐inflammatory cytokines in response to pathogens [40, 41]. Despite these differences, both pDCs and cDCs express the receptor for Ffl3 ligand (Ffl3), and its signaling is essential for their differentiation [42]. Early studies suggested that pDCs and conventional DCs share a common origin from Lin^−^ Flt3^+^CX3CR1^+^CD115^+^ CDPs [43]. Subsequent work demonstrated that pDCs can also arise from common lymphoid progenitors (CLPs), indicating multiple differentiation pathways [44]. More recently, mouse pDCs were shown to derive specifically from pre‐pDC progenitors, defined as Ly6D^+^IL 7Ra^+^Flt3^+^SiglecH^+^CD2^+^CD115^−^ cells, which originate from CLP‐like progenitors [45]. The proposed lymphoid nature of pDCs is further supported by their lymphoid structure, as well as by IgH D‐J gene rearrangement, expression of pre‐TCRα, and CD2 positivity [46, 47]. However, the precise ontogeny of pDCs remains controversial and requires further investigation [38].

The development and maintenance of pDC‐lineage identity depend entirely on the activity of E protein transcriptional factor E2‐2 (TCF4), as its deletion completely abrogates pDC development [48]. An alternative experimental approach has employed transgenic mice expressing diphtheria toxin receptor (DTR) under the control of the blood DC antigen 2 (BDCA‐2) promoter, allowing systemic pDC depletion upon diphtheria toxin administration and enabling functional studies of pDCs in vivo [49].

Functionally, pDCs are best known for their exceptional ability to rapidly produce large amounts of type I interferons (IFNs) in response to viral infection [40]. Beyond this innate immune role, pDCs can also contribute to adaptive immune function, particularly upon activation through Toll‐like receptors (TLRs), when they upregulate MHC II and co‐stimulatory molecules and acquire features resembling cDCs, enabling them to prime and activate naïve T cells [50, 51].

Within the thymus, pDCs are distributed across both the cortex and medulla, with a notable enrichment at the cortico‐medullary junctions (CMJ) [20, 52]. Although, their precise role in thymic biology remains largely unknown, several studies indicate some unique functions in T cell selection and central tolerance. It was demonstrated that pDCs can acquire subcutaneously injected antigens and, in the absence of PRR signals, deliver these antigens to the thymus. Moreover, intravenously injected, antigen‐loaded pDCs were observed to actively enter the thymus and effectively delete antigen‐specific T cells. This migratory capacity is regulated by CCR9 signaling, as genetic ablation of CCR9 prevented pDC entry into the thymus [52, 53]. The extrathymic origin of pDCs, arising from BM progenitors rather than intrathymic precursors, further supports their ability to immigrate into the thymus [34]. Unlike most thymic hAPCs, however, pDCs are inefficient in inducing Tregs, as neither in vitro assays nor in vivo genetic depletion of pDCs revealed a significant impact on thymic Treg generation [19, 24, 54]. This aligns with the observation that, in the absence of microbial stimulation, pDCs function as poor APCs, insufficient to drive robust T cell activation [55]. Interestingly, thymic pDCs can respond to locally produced type I IFNs by upregulating interferon‐stimulated genes (ISGs), MHC II and the chemokine receptor CCR7 [12, 20]. Yet, the functional contribution of those “activated” pDCs in T cell tolerance remains to be determined. Finally, medullary pDCs have been reported to localize in close proximity to Hassall's corpuscles and to constitutively produce IFN‐α, thereby playing a role in T cell maturation [56]. However, subsequent studies employing deep RNA sequencing, quantitative PCR, and IFN‐reporter mice failed to confirm type I IFN expression by thymic pDCs under steady‐state conditions, leaving this proposed role in the thymus under debate [12, 57, 58].

### 2.2 Type 1 Conventional Dendritic Cells (DC1)

As described above, cDCs develop from CDPs in the BM under the control of Flt3 signaling [59, 60]. These progenitors subsequently differentiate into pre‐DC1 or pre‐DC2 lineages [61]. After exiting the BM, these precursors migrate into tissues, where they complete their development and maturation into fully differentiated DC1 or DC2 [61, 62]. In Flt3L‐deficient mice, CDPs and both pre‐DC populations in the BM are severely reduced, resulting in the diminishment of committed DC1 and DC2 cells in peripheral tissues and the thymus [63, 64]. The commitment of DC1 is regulated by several transcription factors, including ID2, NFIL3, BATF3, and IRF8 [65, 66, 67, 68, 69] (Figure 2). Among these, IRF8 plays an essential role as it is required for the generation of pre‐DC1 from CDPs, and its deletion resulted in the complete loss of DC1, which cannot be restored under inflammatory conditions [70]. BATF3‐deficient mice also exhibit a near‐complete absence of DC1 in both peripheral tissues and the thymus; however, pre‐DC1 numbers remain unaffected, and DC1 development can still occur under inflammatory settings [65, 66]. Furthermore, DC1 specification is also dependent on NFIL3‐mediated suppression of ZEB2, which in turn enhances ID2 activity and reinforces DC1 lineage commitment [71].

DC1 are characterized by the expression of the chemokine receptor XCR1, C‐type lectin receptor CLEC9A, as well as CD205 and CD207. In non‐lymphoid tissues, DC1 express integrin αE (CD103) and CD24 [29]. These markers are also applicable for the identification of DC1 in the thymus [72]. Functionally, DC1 are specialized in cross‐presenting antigens to CD8^+^ T cells and in promoting Th1 differentiation [66]. As such, they play a critical role in priming CD8^+^ T cells during antiviral and antitumor immune responses and are an essential part of effective responses to checkpoint blockade therapy [73]. Beyond their role in cytotoxic immunity, DC1 also contribute to maintaining homeostasis in mucosal tissues, such as the lungs and intestine, by sampling microbial antigens and exerting tolerogenic functions [74]. A detailed overview of DC1 functions in immune responses has been comprehensively reviewed elsewhere [29].

In the thymus, DC1 develop intrathymically from immigrating pre‐DC1 precursors [34, 75]. Early studies proposed that DC1s and committed T cells arose from a shared intrathymic progenitor [76, 77]. However, it is now established that DC1 originates from distinct pre‐DC‐related progenitors rather than from CLP [78]. This confusion came from the early experiments showing that both thymic DC1 and committed T cells could differentiate from the DN1 stage of double‐negative thymocytes. Subsequent work revealed that the DN1 compartment is heterogenous, containing DN1a and DN1b subsets that represent early thymocyte progenitors, and the DN1c subset that corresponds to pre‐DC1 precursors. These findings clarified that thymic DC1 arise from the same DC progenitors that give rise to DC1 in the periphery [79, 80, 81]. Pre‐DC1 cells enter the thymus during embryonic development in a CCR7‐dependent manner, giving rise to fully committed DC1 cells that predominantly localize to the thymic medullary region [82]. Interestingly, CCR7 signaling does not appear to regulate DC1 medullary accumulation, as CCR7 deficiency does not affect the DC1 positioning in the medulla, even though the CCR7 ligands are predominantly expressed by mTECs [83, 84]. Instead, DC1 intrathymic localization is governed by the XCR1‐XCL1 axis, with XCL1 preferentially expressed by AIRE^+^ mTECs and NK cells residing in the medulla [85, 86].

The thymic function of DC1 has been primarily attributed to their exceptional ability to acquire and cross‐present self‐antigens produced by mTECs [19, 87, 88] (Figure 1). Self‐antigens expressed by mTECs can be presented to developing T cells either directly by mTECs themselves or indirectly through DCs [22, 89]. Notably, studies using fluorochromes selectively expressed by AIRE^+^ mTECs to mimic TSA expression demonstrated that these antigens were almost exclusively captured by XCR1^+^ DC1s [87, 88]. In contrast, fluorochromes uniformly expressed across all TECs were acquired by both DC1 and DC2 subsets with comparable efficiency, suggesting that the superior ability of DC1 to capture antigen from AIRE^+^ mTECs is driven by DC1‐specific mechanisms [18, 88]. One such mechanism involves the XCR1‐XCL1 axis, in which DC1s expressing the chemokine receptor XCR1 are recruited toward AIRE^+^ mTECs producing XCL1 [85]. More recent evidence indicates that within the XCR1^+^ DC1 pool, it is predominantly the activated CCR7^+^ subset (aDC1) that efficiently captures and presents mTEC‐derived antigens [72, 88]. However, it remains unclear whether aDC1s possess intrinsic features that enhance antigen presentation, or whether antigen capture itself contributes to DC1 activation within the thymus [90].

In addition to chemokine‐mediated recruitment, DC1s uniquely express the scavenger receptor CD36, which binds phosphatidylserine presented on apoptotic cells. CD36 has been shown to facilitate the transfer of surface antigens from apoptotic mTECs to DC1s, enabling their presentation on MHC II molecules [87]. More recently, the tight junction protein Claudin‐1 (CLDN1), was identified as a regulator of mTEC‐DC1 interactions, thereby influencing antigen transfer. Interestingly, DC1‐specific loss of CLDN1 impaired DC1 maturation and disrupted Treg selection [91]. This is consistent with a seminal study comparing TCR repertoires of Tregs in wild‐type, AIRE‐deficient, and BATF3‐deficient mice, which collectively demonstrated that DC1s are the principal DC subset presenting AIRE‐dependent mTEC‐derived antigens for Treg selection [19]. The role of thymic DC1 in supporting Treg generation is further supported by various in vitro studies, which show that co‐culture of DC1s with CD4^+^ thymocytes induces Treg differentiation [84, 92]. Interestingly, this process was found to be regulated by CD27‐CD70 signaling, highlighting the importance of co‐stimulation in DC1‐dependent Treg induction [92].

### 2.3 Type 2 Conventional Dendritic Cells (DC2)

Peripheral DC2 commonly express CD11b and CD172α (SIRPα) [29] (Figure 2). Their terminal differentiation was shown to be influenced by the transcription factor IRF4, although IRF4 regulates only a limited set of genes primarily linked to DC2 activation and migratory capacity. Consequently, IRF4 deficiency leads to only a partial reduction of DC2 numbers in peripheral tissues [93, 94]. In addition, DC2 development is also shaped by transcription factors KLF4 and IRF2 as their deletion alters the DC2 numbers and phenotype in the spleen [95, 96]. More recently, the triple mutation in NFIL3‐C‐EBP binding site was shown to completely abolish *Zeb2* expression in myeloid, but not lymphoid, progenitors, resulting in loss of pre‐DC2 specification and consequently blocked DC2 development [97].

Functionally, DC2 are essential for the effective activation of CD4^+^ T helper (T_H_) cells' immune responses and particularly important for generating T_H_2 responses against helminth infections [29, 97, 98]. However, the function of DC2 has also been implicated in the activation of CD8^+^ cytotoxic T cell (T_C_) responses, specifically in tumor settings, where interferon (IFN)‐stimulated DC2 activate CD8^+^ T cells by presenting intact tumor‐derived p:MHC I complexes via the process of “MHC‐dressing” [99]. Collectively, these findings indicate that peripheral DC2 contribute to diverse immune functions, supporting both CD4^+^ T_H_ and CD8^+^ T_C_ responses.

In association with the diverse functions of DC2, these cells exhibit substantially higher internal heterogeneity compared to DC1 (Figure 3). Initially, two subgroups of DC2 were defined based on their differential developmental requirements for NOTCH2 or KLF4 activity [95, 100, 101]. NOTCH2‐dependent DCs were found to express high levels of endothelial cell‐selective adhesion molecule (ESAM) in the spleen and CD103 in the intestinal lamina propria [100]. In contrast, NOTCH2‐independent, KLF4‐dependent DC2s were strongly labeled in *Cx3cr1* and *Ccr2* reporter mice, as well as in the *Lyz2* fate mapping system [95, 100]. KLF4‐dependent DC2s were also shown to express CD301b and PD‐L2 and to highly respond to type 2 cytokine stimulation [98, 102]. Subsequently, a study using *Tbx21* and *Rorc* reporter mice identified T‐bet^+^ cDC2A and RORγt^+^ cDC2B populations, respectively. Notably, T‐bet^+^ cDC2A cells were enriched for ESAM^+^ DC2 cells, linking cDC2A to the NOTCH2‐dependent DC2s [103]. However, the expression of T‐bet and RORγt at both mRNA and protein levels has not been conclusively confirmed, with the exception of a small population of RORγt^+^ APCs that represent a distinct subset of lymphoid cell types rather than DCs [104, 105]. The origin, heterogeneity and potential functions of these cells have been reviewed in detail elsewhere [106]. An additional study proposed that KLF4‐dependent DC2 correspond to the cDC2B subset [107].

Furthermore, several groups have described a subpopulation of peripheral DC2 cells that display many pDC‐like features, including the expression of and dependence on the transcription factor TCF4 [107, 108, 109]. These cells, referred to as pDC‐like cells or transitional DCs (tDC) share greater developmental similarity with pDCs than with classical DC2 cells and were shown to further differentiate into ESAM^+^ cDC2A cells [109]. This finding was supported by the study using a CD300c lineage‐tracing approach, which demonstrated that lymphoid‐derived pDC‐like precursors give rise to a subset of DC2 cells, primarily ESAM^+^ cDC2A [110]. More recently, two distinct pre‐DC2 populations were identified in BM: (1) SiglecH^+^ CD115^−^ cells, representing pDC‐like precursors that give rise to tDCs and subsequently DC2A cells in a TCF4‐dependent manner, and (2) SiglecH^−^ CD115^+^ cells, representing conventional pre‐DC2s that give rise to CD11b^+^ DC2B in a KLF4‐dependent manner [111]. Together, these findings confirm that the splenic DC2 population comprises two developmentally distinct lineages representing tDC‐related ESAM^+^ CD11b^−^ DC2A cells and conventional CD11b^+^ DC2B cells [110, 111, 112].

DC2 cells are present in the thymus and exhibit the classical phenotype of Sirpα^+^ CD11b^+^ CD8α^−^ cells [34]. Unlike DC1, which originate through intrathymic differentiation, DC2 are thought to migrate into the thymus from the periphery as differentiated cells that subsequently integrate into the thymic parenchyma [21, 34]. However, several studies have reported that only a minority of thymic DC2 have an extrathymic origin and are capable of immigrating into the thymus later in life [21, 113]. It remains unclear what proportion of thymic DC2 represent true immigrating cells and whether these DC2 undergo any form of differentiation within the thymic environment.

As in the peripheral tissues, thymic DC2 are considerably more heterogeneous than DC1. Historically, several studies have suggested the existence of various subpopulations of SIRPα^+^ DC2 in the thymus. Adoptive transfer experiments, in which peripheral DCs we introduce into the mouse bloodstream, revealed two populations of DC2 capable of migrating into the thymus: CD8a^−^ CD11b^+^ and CD8a^−^ CD11b^−^ [21]. Subsequent studies indicate that SIRPα^+^ DC2 include a subpopulation of cells characterized by high expression of CX3CR1. These cells have been described as monocyte‐derived DCs, CX3CR1^+^ DCs, or CX3CR1‐dependent transendothelial DCs (TE‐DCs) [114, 115, 116]. Additionally, a population of CD301b^+^ DC2 cells was identified, representing approximately 40% of thymic SIRPα^+^ cells. However, these are thought to reflect type 2 cytokine activation rather than a distinct subpopulation [113].

Recently, we and others combined single‐cell transcriptomics with lineage‐defining mouse models to investigate the heterogeneity of SIRPα^+^ cells in the thymus [20, 36, 113, 117, 118]. Thymic SIRPα^+^ DCs (e.g., CD11c^+^MHCII^+^ Sirpα^+^CD8α^−^) were shown to include populations of monocyte‐derived cells, defined by the expression of Ly6C, CD64, and F4/80, as well as by *Ms4a3*‐dependent fate‐mapping, which specifically labels cells originating from granulocyte‐monocyte progenitors (GMPs) [20, 119] (Figure 3). Interestingly, these monocyte‐derived cells account for approximately 15% of thymic SIRPα^+^ DCs [20]. In addition, SIRP*α* ^+^ DCs comprise classical DC2, defined by the expression of CD11b and conventional DC2‐associated markers such as the inhibitor of DNA binding (*Id2*), lymphotoxin‐b (*Ltb*), and *Klf4* [20, 113]. Moreover, the thymic SIRPα^+^ DCs also consist of cells that show a clear dependency on TCF4, and express markers previously associated with splenic tDCs [20, 109]. Notably, these cells can be identified in the thymus by high expression of CX3CR1, with low or absent expression of CD11b, thus corresponding to CD11c^+^MHCII^+^ SIRPα^+^CX3CR1^+^CD11b^−^. This suggests that the previously defined population of CX3CR1^+^ DCs includes not only monocyte‐derived cells but also a prominent subset of thymic tDCs. Interestingly, the subclassification and nomenclature of splenic DC2 into DC2A and DC2B cannot be directly applied to the thymus, as neither ESAM nor other NOTCH2‐dependent genes (e.g., *Dtx1*, *Hes1*, and *Clec4a4*) are expressed in thymic DC2s. Therefore, although two developmentally distinct DC2 populations were identified, they were more accurately defined as tDCs and DC2s to reflect their unique developmental origin and phenotypic features in the thymus [20, 118].

Understanding the DC2 function in the thymus remains challenging, as no genetic tools are available to selectively ablate DC2 as a whole population (the Zeb2 mutant mice described above also lack monocytes). Moreover, given the substantial heterogeneity within SIRPα^+^ DC2, dissecting the role of individual subtypes is even more complex. Nonetheless, several studies have proposed various thymus‐related functions of the total SIRPα^+^ DC2 population. In vitro experiments and models employing neo‐self‐antigens expression in the thymus demonstrated that SIRPα^+^ DC2s can drive Treg selection as well as activate transgenic CD8^+^ and CD4^+^ thymocytes [24, 84, 120]. This was further supported by a study implying that Treg selection against prostate‐specific self‐antigens requires DC2, as no differences were observed in DC1‐depleted mice [54]. Another key function of DC2 relates to their described ability to immigrate into the thymus and transport peripheral antigens. Intravenous protein injection experiments indicated that DC2 can capture the circulating antigens and present them to developing T cells [21]. This function is particularly associated with a subpopulation of DC2 termed TE‐DCs, which reside in close proximity to thymic microvessels and specialize in sampling blood‐born antigens [115]. In addition, DC2 exhibit a superior capacity to capture free antigens compared to other thymic APCs [121, 122]. This enables them not only to acquire soluble antigens, but also to cross‐present self‐antigens obtained from mTECs or other DCs [88, 122]. More specific insight into DC2 function has come from a partial ablation study using the CD301b‐depletion system. Selective depletion of CD301b^+^DC2 altered the deletion of CD4^+^ T cells, suggesting a critical role for this subset in clonal deletion as well [113].

### 2.4 Transitional Dendritic Cells (tDC)

As described in the previous chapter, thymic Sirpα^+^ DC2 cells include a previously unappreciated subpopulation of CX3CR1^+^ tDCs [20]. tDCs were first identified using high‐dimensional single‐cell approaches in human blood and were found to be conserved across humans and mice [62, 108, 123, 124]. In humans, tDCs are defined by the expression of AXL and SIGLEC6 and have been referred to as ASDCs (AXL/SIGLEC6 DCs) or AXL^+^ DCs [62, 123]. However, these markers are not conserved in mice, preventing their identification using the same criteria [108]. Consequently, the term ‘transitional DCs’ has been adopted to emphasize their transcriptomics, phenotypic and functional features which span characteristics of both pDC and DC2, representing a transitional state between the two lineages [108, 109]. This transitional spectrum allows the identification of two major subsets of tDCs: CD11c^lo^MHCII^lo^ tDCs (tDC^lo^) which are more similar to pDCs, and CD11c^hi^MHCII^hi^ tDCs (tDC^hi^), which resemble DC2 [108, 109]. Accordingly, tDC^lo^ represent already described pDC‐like cells [107], whereas tDC^hi^ were attributed to noncanonical DCs [125]. Recently it was described that both tDC^lo^ and tDC^hi^ cells share transcriptional and developmental features with pDCs and require TCF4 for their differentiation. Lineage‐tracing studies in mice using human CD2^Cre^ (hCD2^Cre^) or CD300c^Cre^ models further confirmed their emergence from pDC‐related progenitors [109, 110]. Evidence from the spleen also suggests a developmental trajectory in which tDC^lo^ cells give rise to tDC^hi^, that upregulate several DC2‐associated transcription factors such as ZBTB46 and IRF4. Interestingly, tDC^hi^ cells can then convert into ESAM^+^DC2s via an intermediate CD11b^−^ DC2 stage. Notably, this transition involves distinct transcriptional factors. While TCF4 is essential for the early development of tDCs from pDC‐related progenitors, IRF4 is required for the transition of tDC^hi^ into ESAM^+^ DC2 [109]. Notably, a subset of ESAM^+^ DC2 exhibits immunoglobulin heavy chain (IgH) expression, a characteristic shared with pDCs and tDCs, both of which display IgH D‐J gene rearrangements together with the expression of pre‐TCRa [109, 126].

Functionally, tDCs resemble classical DC2 cells, as they respond to toll‐like receptors (TLRs) stimulation, which strongly reprograms their transcriptional profile toward the upregulation of various genes associated with pro‐inflammatory responses. This includes changes in antigen processing and presentation, as well as increased production of various proinflammatory molecules [107]. Notably, tDCs can produce high levels of interleukin‐1b (IL‐1b) in response to virus stimulation, which appears to play a protective role during immune responses [109]. Nevertheless, the specific function of tDCs in various immune responses remains to be fully determined.

Historically, analysis of IgH D‐J gene rearrangements in thymic DCs revealed not only the presence of pDC‐related progenitors, but also fully differentiated DCs with lymphoid features [127]. The existence of such progenitors was further supported by the identification of pDC‐like cells (corresponding to tDC^lo^ cells) within the thymus. These cells were characterized as BST2^+^SiglecH^+^ cells, expressing ZBTB46^GFP^, and displaying positivity for CD14 and CX3CR1 [107].

In our recent study, single‐cell transcriptomic analysis of thymic CD11c^+^ and CD11b^+^ cells revealed a distinct population of CX3CR1^+^ tDC that clusters between thymic pDCs and DC2, while simultaneously upregulating classical splenic tDC‐associated genes, such as *Tcf4*, *Cd209e*, *Ngfr*, or *Cd14*. By flow cytometry, thymic tDC could be clearly defined as TCF4^+^CX3CR1^+^CD14^+^ cells lacking the canonical pDC markers SiglecH and B220, and notably also negative for CD11b. Lineage‐tracing demonstrated that thymic tDCs were not labeled in the *Ms4a3*‐dependent fate‐mapping system but were efficiently marked in the *hCD2* ^*Cre*^ model. Furthermore, genetic ablation of *Tcf4* specifically in DCs resulted in a profound loss of both thymic pDC and tDC populations, supporting their shared developmental origin [20]. In contrast to splenic tDCs, which under specific conditions can further differentiate into ESAM^+^ DC2A cells through NOTCH2‐dependent gene programs, such ESAM^+^ DC2A cells were absent from the thymus [20, 109]. This aligns with the observation that conditional deletion of *Notch2* reduces splenic DC2 numbers without affecting thymic DC2s [128]. Finally, mice carrying triple mutations in *Zeb2* enhancer exhibited near‐complete loss of both thymic DC2s and tDCs, while thymic pDCs remained unaffected, highlighting the unique developmental pathway of tDCs [20, 97]. However, the precise characteristics of thymic tDC^lo^ and tDC^hi^ subsets, along with their developmental relationships and regulatory mechanisms, remain to be further refined.

Together with their intrathymic regulation, the function of tDCs in the thymus remains to be fully elucidated. Nonetheless, our recent study has shown that tDCs are uniquely localized near thymic microvessels or within the perivascular space, enabling them to sample the antigens from the bloodstream. Supporting this, tDCs were efficiently labeled by intravenously injected anti‐CD11c monoclonal antibodies, confirming that a substantial fraction of these cells is indeed exposed to the blood‐circulating antigens. These observations led to the conclusion that thymic tDCs correspond to the previously described TE‐DCs, which are responsible for presenting blood‐born peripheral antigens to developing T cells [20].

### 2.5 Monocyte‐Derived Cells and Macrophages

As mentioned previously, the thymic SIRPα^+^ DC pool includes the population of monocyte‐derived cells that adopt the phenotype of classical DCs [20, 36]. In addition, the thymus harbors both resident tissue macrophages (RTMs) and monocyte‐derived macrophages [36]. It is well established that the first wave of hematopoietic progenitors arises during early embryonic development, giving rise to yolk‐sac derived RTMs [129, 130]. These cells colonize developing tissues and support organogenesis and tissue homeostasis [130]. At later stages of embryonic development, erythro‐myeloid progenitors and hematopoietic stem cells (HSCs) can similarly give rise to monocytes and macrophages, which further support tissue development and homeostasis [131, 132]. In adulthood, all hematopoietic cells are derived from HSCs, that give rise to CMPs and granulocyte‐monocyte progenitors (GMPs), both of which can differentiate into monocytes and monocyte‐derived cells through cMoPs [37, 133]. A detailed overview of monocyte and macrophage development, as well as their tissue‐specific functions, has been comprehensively reviewed elsewhere [37].

Functionally, the RTMs are long‐lived cells responsible for clearing cellular debris, regulating tissue repair and maintenance, preserving tissue integrity, and providing the first‐line defense against pathogens [134]. Conversely, monocytes and monocyte‐derived cells are relatively short‐lived and continuously replenished cells, that upon activation through PRRs signaling, acquire the phenotype and functions of classical DCs, acting as potent antigen‐presenting cells capable of activating naïve T cells and initiating adaptive immune responses [135, 136].

Although RTMs have long been known to exist in the thymus, their thymus‐specific functions remain incompletely understood. Thymic macrophages are best recognized for their role in clearing apoptotic thymocytes [137]. This phagocytic activity had also been described to be essential for proper negative selection of T cells, as depletion of thymic phagocytes leads to increased survival of thymocytes that received strong TCR signals [138]. Notably, the scavenger receptor TIM4 has been shown to play a central role in this process [138, 139]. More recently, thymic macrophages were found to be heterogeneous, comprising two major populations defined by the expression of TIM4 and CX3CR1, and dependent on the transcription factor *Nr4a1* [36, 140]. However, the study of thymic macrophages has been complicated by the lack of consensus on their characterization and their phenotypic overlap with thymic DCs. For instance, the classical macrophage marker F4/80 is also expressed by monocytes, thymic eosinophils, and a subset of cells with potent antigen‐presentation capacity [36, 141]. Similarly, the conventional DC marker CD11c is co‐expressed with F4/80 on certain DC‐like cells in the thymus [20, 142].

Our recent data suggest that the thymic monocytes/macrophages compartment, defined by the expression of transcription factors *Csf1r* and *Mafb*, consists of four major populations: monocytes, monocyte‐derived DCs (moDCs) and two populations of macrophages [20] (Figure 2) Interestingly, these macrophage subsets can be distinguished by TIM4 expression, separating them into TIM4^+^ and TIM4^−^ macrophages [20]. By comparing their transcriptional and protein profiles with previously published findings, we found that TIM4^+^ cells correspond to yolk sac‐derived RTMs, whereas TIM4^−^ cells represent CX3CR1^+^ macrophages derived from adult HSCs [36]. This distinction was further confirmed using Ms4a3^Cre^ROSA26^tdTomato^ mice, in which recombination occurs only in monocytes, moDCs, and TIM4^−^ macrophages, confirming their monocyte origin [20, 119]. Based on this, we referred to the TIM4^−^ subset as monocyte‐derived macrophages (moMACs). Interestingly, both moDCs and moMACs exhibit upregulation of CD11c and MHC II expression, along with high expression of co‐stimulatory molecules, making them transcriptionally and phenotypically like thymic DCs [20, 36].

Functionally, next to their role in apoptotic cell clearance, thymic macrophages have been shown to efficiently present antigens and activate naïve T cells, confirming that the moMAC subset of thymic macrophages represents potent antigen‐presenting cells [36]. Moreover, thymic moDCs are recruited into the thymus by TLR9‐stimulated mTECs and contribute to enhanced Treg selection following TLR activation [114]. In addition, thymic macrophages sustain stable IL‐4 production by thymic iNKT cells, thereby promoting the maturation of thymic DC2s into CD301b‐expressing cells [113, 143]. Together, these findings highlight the diverse thymic roles of monocytes/macrophages, ranging from the clearance of apoptotic thymocytes and antigen presentation to shaping the thymic microenvironment through the regulation of IL‐4 production.

## 3 Homeostatic Maturation of Thymic DCs, Monocytes and Macrophages

**FIGURE 4:**
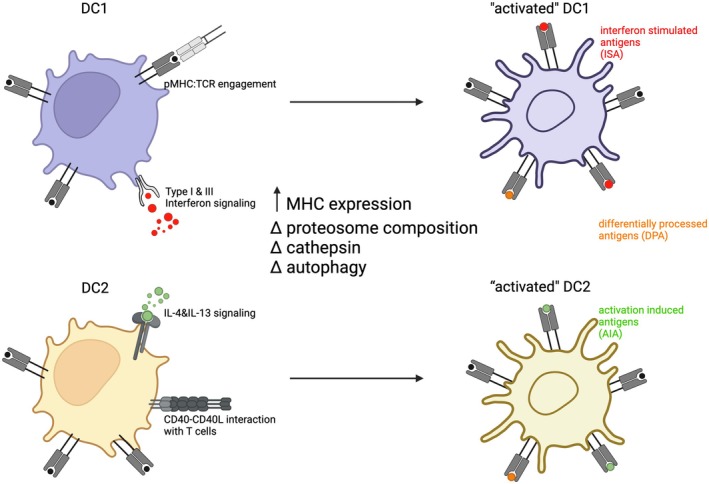
Homeostatic maturation of thymic DCs is essential for central tolerance. Within the thymus, DCs undergo a process of homeostatic maturation that is required for their tolerogenic function. In their immature state, DC1 and DC2 display basal self‐peptidomes (black). Upon receiving maturation cues, DCs become activated, enabling them to effectively mediate clonal deletion or promote Treg differentiation. DC1 respond predominantly to Type I&III interferon signaling, broadening their peptidome to include inflammation stimulated antigens (ISAs), thereby enhancing clonal deletion. By contrast, DC2 predominantly respond to type 2 cytokine–driven signals (IL‐4 & IL‐13 signaling), remodeling their peptidome to incorporate activation induced antigens (AIAs) (green), supporting CD4^+^ T cell tolerance and Treg induction. This maturation‐linked reshaping of the antigenic repertoire ensures that thymic DCs present a comprehensive spectrum of self‐antigens, safeguarding central tolerance under both steady‐state and stress‐associated conditions. Homeostatic maturation also causes shared changes in DCs, such as upregulation in MHC expression, changes in compositions of endosome, cathepsin, etc. These changes in APCs' antigen processing and presentation abilities lead to further remodeling of their peptidomes to incorporate differentially processed antigens (DPAs) (orange).

An interesting feature of the thymic hAPC compartment is that they acquire characteristics of highly activated cells, marked by the overexpression of MHC II, CD40, and co‐stimulatory molecules CD80 and CD86 [20, 72, 88, 117, 144] (Figure 4). This activated phenotype is primarily observed in DCs and monocyte‐derived cells, giving rise to aDCs, moDCs and moMACs [20]. Notably, thymic B cells are also intrathymically licensed to undergo class‐switching and become activated B cells, which are highly effective antigen‐presenting cells regulating clonal deletion and Treg selection [11, 145]. A comprehensive overview of thymic B cells licensing and function has been provided elsewhere [146].

Several studies have demonstrated that aDCs localize predominantly within the thymic medulla. Compared with their non‐activated counterparts, these cells are more efficient at cross‐presenting mTEC‐derived antigens to developing T cells, thereby establishing central tolerance [72, 88, 147]. This strongly suggests that intrathymic DC activation plays an essential role in T cell selection.

The thymic DC pool comprises homeostatically activated subsets characterized by the upregulation of CCR7, CD63, and MHC II [20, 72, 113]. These cells are thought to represent the activated counterparts of DC1 and DC2, respectively. In our recent publication, we demonstrated that aDCs form a continuum of cells ranging from early activated populations, co‐expressing CCR7 together with DC1‐ or DC2‐lineage‐defining molecules such as XCR1 or SIRPα, to fully matured late aDCs, which substantially downregulate transcriptional and protein characteristics of their respective DC lineage. Our study further shows that both thymic aDC subsets are activated subtypes of DC1 and DC2, as evidenced by their dependence on either the DC1‐lineage defining BATF3‐deficient mouse model or the DC2 depleting ZEB2 triple‐mutant mouse [20]. Interestingly, despite their distinct ontogeny, aDC1 and aDC2 display highly similar transcriptional profiles, suggesting that they undergo a universal DC maturation program that converges into a functionally unified APC population within the thymus [20, 113]. In addition, this transcriptional similarity is also observed in the previously mentioned moDC and moMAc populations, which represent thymically activated subsets of monocyte‐derived cells [20].

### 3.1 Maturation Regulated by Thymic Sterile Inflammation

The thymus exhibits constitutive activation of multiple inflammatory pathways, including TLR signaling and interferon production by mTECs, and type II cytokine production by NKT cells [8, 12, 113, 114]. As described above, this sterile inflammatory environment broadens the repertoire of self‐antigens to include ISAs and promotes central tolerance to inflammatory self. Moreover, it profoundly shapes the activation of thymic hAPCs, both consolidating their tolerogenic properties and diversifying their pMHC ligandomes to include modified self‐antigens associated with peripheral inflammatory contexts (i.e., inflammation‐associated self‐antigens, ISAs) and activation (i.e., activation‐induced antigens, AIAs) [7, 9].

Remarkably, mTECs constitutively produce type I and type III interferons (IFNs). The expression of these IFNs is regulated by AIRE and begins early in life and peaks around 3 weeks of age [12, 57, 58]. Nearly all thymic hAPCs respond to IFNs by upregulating interferon‐stimulated genes (ISGs) and dramatically shaping their transcriptomes toward inflammatory states [12]. This raises the question of whether IFN signaling drives the activation program of thymic DCs and monocyte‐derived cells. Indeed, DC1 maturation is highly dependent on type III IFN signaling, as mice lacking type III IFN receptor show a marked reduction in aDC1. Similarly, the maturation of monocyte‐derived cells is strongly influenced by both type I and type III IFNs, since moDCs and moMACs are dramatically reduced in mice lacking receptors for these IFNs. By contrast, DC2 maturation is not affected by IFN‐signaling deficiency, indicating that despite their huge transcriptional similarities, aDC1 and aDC2 rely on distinct signals for intrathymic maturation [12, 20].

A substantial fraction of thymic DC2 express the activation marker CD301b and display an activated phenotype. This population is further defined by a transcriptional signature associated with exposure to type 2 cytokines, including IL‐4, IL‐5, IL‐9 and IL‐13. Notably, CD301b^+^ DC2s are dramatically reduced in mice lacking IL‐4 receptor signaling, which abrogates the IL‐4 and IL‐13 responses. These findings suggest that type 2 cytokine signaling, much like IFN signaling, not only enhances the activation of thymic DC2s but also shapes their pMHC ligandome to present self‐antigens linked to type 2 inflammatory responses [113].

Importantly, the constitutive production of these inflammatory mediators in the thymus represents a sterile form of inflammation, as their expression is independent of PRR and cytosolic nucleic acid receptor signaling and unaffected by a germ‐free (GF) environment [12]. This highlights that, although the transcriptomes of aDC1, aDC2, and activated monocyte‐derived cells strongly resemble those of peripherally activated counterparts, intrathymic maturation proceeds normally in GF mice as well as in mice deficient in the PRR signaling adaptors MyD88, MAVS, and TRIF [12, 72, 144].

### 3.2 Maturation Regulated by Crosstalk With Thymocytes

Another layer of thymic hAPC maturation depends on the crosstalk between post‐positive selection thymocytes and DCs. Studies have shown that the numbers of aDC1, aDC2 and monocyte‐derived cells decline dramatically in mice lacking single‐positive (SP) T cells, indicating that these thymocytes provide key signals for hAPCs maturation [20, 144, 147, 148]. In particular, CD40‐CD40L signaling plays an essential role in thymic DC maturation. CD40L is expressed predominantly by CD4^+^ thymocytes, and their cognate interaction with DCs drives DC activation [147, 148]. Consistent with this, CD40 deficient BM‐derived cells exhibit a marked reduction in the frequency of both aDC1 and aDC2 subsets in the thymus [144]. These findings suggest that CD4^+^ thymocytes play an essential role in thymic DC activation. This was further supported by evidence showing that the absence of MHC II expression on DCs leads to a marked reduction in aDC numbers [144]. It has also been proposed that cognate interactions with CD8^+^ thymocytes may promote thymic DC activation, potentially through a CD40L‐independent mechanism [148]. However, more recent experiments using *β*2m‐deficient mice, which lack MHC I, demonstrated no reduction in thymic aDC numbers. This indicates that CD8^+^ thymocytes are dispensable for thymic DC activation [147].

In our recent publication we enumerated the total numbers of aDC1 and aDC2 in CD40L‐deficient mice and surprisingly, only the population of aDC2 showed significant reduction, suggesting that CD40‐CD40L signaling is primarily essential for DC2 maturation [20]. Moreover, the CD40L deficiency does not seem to be involved in the maturation of thymic moDC and moMACs as no specific reduction was observed in these populations. However, both activated subsets of monocyte‐derived cells were markedly reduced in mice lacking SP thymocytes in TCRα‐deficient mice, suggesting the indirect role of T cells in thymic monocyte‐derived cell activation [20].

These findings suggest that crosstalk with thymocytes not only enhances the activation of thymic DC2s but also shapes their pMHC ligandome. In particular, it promotes the presentation of activation‐induced self‐antigens (AIAs), and differentially processed antigens (DPA), reflecting activation‐induced changes in antigen processing and presentation machinery (Figure 4).

## 4 Division of Labor Among Thymic DCs in Central Tolerance

For splenic DCs, it has been established, that DC1 and DC2 differ in their antigen processing and presentation machinery. DC1 excel at cross‐presenting exogenous antigens to CD8^+^ T cells, whereas DC2 are more efficient at presenting endocytosed antigens to CD4^+^ T cell [149]. This functional dichotomy also appeared to apply in vitro, as thymic DC1 more effectively stimulated CD8^+^ T cells through cross‐presentation, while DC2 preferentially stimulated CD4^+^ T cells via MHC II loading [122]. However, in vivo studies using BATF3‐deficient mice, which lack DC1 cells, revealed that DC1 depletion had essentially no impact on the CD8^+^ T cells repertoire [66, 150]. This suggests that DC1‐mediated cross‐presentation is disposable for CD8^+^ T cells tolerance. Similarly, DC1 depletion had no major effect on CD4^+^ T cell repertoire, with rates of negative selection and Treg generation remaining comparable between BATF3‐deficient and sufficient mice [54]. However, when specifically examining AIRE‐dependent antigens, it was clearly shown that DC1 are the main thymus DC subset involved in AIRE‐dependent antigen presentation; DC1 were shown to be the principal thymic subset responsible for presenting these antigens for Treg induction [19, 87]. This conclusion was further supported by the observation that MHC II‐deficient DC1 mice exhibited a significant reduction in the polyclonal thymic Treg population [91]. Together, these findings indicated that DC1 represent a key DC type important for thymic Tregs induction.

As mentioned above, a system that selectively depletes thymic DC2 as a whole is still not available. Based on current knowledge, ZEB2 triple‐mutant mice, which completely lack the thymic DC2 population, could be used to assess the specific function of DC2 in the thymus [20, 97]. However, TCR repertoire analysis in these DC2‐deficient mice has not yet been performed. Moreover, ZEB2 triple‐mutant mice also lack thymic moDCs and moMACs, indicating that achieving specific DC2 depletion in the thymus remains a significant challenge [20].

Reling on in vitro experiments and models employing neo‐self‐antigens expression in the thymus it has been demonstrated that DC2s can drive Treg selection as well as activate transgenic CD8^+^ and CD4^+^ T cells [24, 84, 120]. However, partial depletion of thymic DC2 expressing CD301b significantly impaired the deletion of polyclonal CD4^+^ T cells, without affecting Treg generation [113]. Interestingly, it was also reported that an increase in the thymic DC2 population markedly enhanced T cell clonal deletion at the population level, as measured by cleaved caspase‐3 positivity [12, 113]. These findings suggest that thymic DCs play non‐redundant roles in T cell selection, with DC1 primarily supporting Treg induction and DC2 contributing to clonal deletion of conventional T cells. Nevertheless, these results remain preliminary, and further studies are required to reconcile and extend these observations.

The distinctive features of DC1 and DC2 likely predispose them to access and present distinct pools of self‐antigens. Several studies have reported that DC1 is significantly more efficient than DC2 in acquiring self‐antigens derived from mTECs [87, 88, 147]. Notably, it has been suggested that different DC subtypes ‘pair’ with specific subtypes of TECs to sample antigens from distinct sources. In this model, DC1 and aDC1 preferentially acquire antigens from AIRE^+^ mTECs, whereas DC2 more commonly ‘pairs’ with mTEC^Low^ or mimetic cells [88]. This dichotomy may underline their functional specialization, as the presentation of TSA‐like antigens by DC1 tends to promote Treg induction, whereas the presentation of more abundant self‐antigens by DC2 is more often linked to clonal deletion [151]. Additionally, thymic moDC have been shown to acquire antigens not only from mTECs and mimetic cells (such as microfold cells) but also from other thymic DCs, equipping them to present a diverse array of self‐antigens for both Treg induction and clonal deletion [6, 88]. Moreover, the presentation, of peripheral or blood‐born antigens by thymic pDCs, DC2 and the newly described tDCs adds another layer of complexity, as strategic positioning, cellular characteristics, and capacity to immigrate into the thymus from peripheral tissues may profoundly influence their roles in Tregs selection and clonal deletion [20, 21, 52, 115].

## 5 Thymic Immigration

As mentioned above, thymic DC2 cells were initially thought to migrate into the thymus from the periphery as differentiated cells that subsequently integrate into the thymic parenchyma [34]. However, studies using parabiotic mouse models, which share blood circulation, revealed that only about 10%–15% of SIRPα^+^ DCs are exchanged between parabionts [21, 113]. This finding indicates that only a minority of DC2 can migrate into the thymus from the periphery. Functionally, it has been suggested that these immigrating DCs transport antigens from peripheral tissues into the thymus. For example, administration of fluorescent isothiocyanate (FITC) into the dorsal skin resulted in the detection of FITC^+^ SIRPα^+^ DCs in the thymus, suggesting that DCs may migrate from the skin to the thymus [21]. This phenomenon of intrathymic immigration was further confirmed using photoconvertible transgenic mice, in which all cells express GFP, that irreversibly converts to RFP after light exposure. Strikingly, photoconversion of the mouse caecum led to the appearance of RFP^+^ cells within the thymus, confirming that DCs can migrate to the thymus from various peripheral tissues [116].

The migration of DC2 into the thymus has been shown to depend on various adhesion molecules and chemokine receptors. The entry of DC2 into the thymus is regulated by P‐selectin and the integrin VLA‐4, which binds to VCAM‐1. Notably, blocking antibodies against integrin VLA‐4 were shown to inhibit DC2 migration into the thymus [21]. Chemokine receptor CCR2 plays an important role in the immigration of thymic SIRPα^+^ DCs, as CCR2 deficiency results in reduced frequencies of thymic DC2s. Interestingly, the CCR2 ligand, expressed by endothelial cells and TECs, is enriched in the perivascular space, where thymic SIRPα^+^ DCs are also found [121, 152]. However, CCR2 depletion also causes a marked accumulation of DC2s in the BM and a reduction of these cells in the blood, suggesting that CCR2 is more critical for regulating DC2 homeostasis in the circulation rather than directly mediating thymic immigration [121, 153].

More recently, it was reported that CX3CR1^+^ DC2 migrate from the small intestine to the thymus early in life, where they present commensal‐derived antigens and drive the expansion of commensal‐specific T cells [116]. Interestingly, CX3CR1 deficiency did not impair DC2 migration into the thymus, but instead, the migration of CX3CR1^+^ DC2 was shown to be regulated by CCR5 signaling [115, 116]. Although CX3CR1 itself is not required for thymic DC2 entry, it is essential for the accumulation of CX3CR1^+^ DC2 in the perivascular space and their transendothelial localization, which enables the cells to sample and present blood‐born antigens to developing T cells [115]. In our recent work, we identified a population of CX3CR1^+^ tDCs that share a developmental origin with pDCs. These cells efficiently bound intravenously injected CD11c‐PE antibody, localized near thymic microvessels, and appeared in the thymus only at later time points after weaning [20]. These findings linked the previously described CX3CR1^+^ DC2 population with CX3CR1^+^ TE‐DCs and CX3CR1^+^ tDCs, suggesting that they may represent a single unified subset (Figure 2). This also raises the possibility that the perivascular and transendothelial localization of these cells reflects a transient migratory state before their full integration into the thymic parenchyma. Additionally, pDCs have been shown to migrate into the thymus in a CCR9‐dependent manner and to present antigens to developing T cells for clonal deletion [52]. However, since thymic pDCs have not been described as potent APCs, and given their developmental and phenotypic similarity to tDCs, we hypothesize that CCR9‐dependent immigrating pDCs may in fact represent a subpopulation of thymic tDCs [20, 55]. Additional experiments will be required to further evaluate and reconcile these similarities with the published data. Moreover, TLR9 stimulation of mTECs was shown to induce an influx of thymic moDC into the medullary region of the thymus and to enhance the transfer of mTEC‐derived antigens to these DCs. TLR9 signaling increased the expression of several chemokines, among which CCR5‐dependent signaling appeared to be essential for the entry of moDCs into the thymus [114]. Nevertheless, further studies are required to fully elucidate the ontogeny, precise characterization, and functional significance of thymic DC immigration for T cell selection.

## 6 Conclusion and Future Perspective

The thymic dendritic cell compartment has emerged as a complex and heterogeneous system that plays essential roles in establishing central tolerance and shaping the T cell repertoire. The traditional binary classification of thymic DCs into DC1 and DC2 subsets significantly underestimates the true diversity of thymic antigen‐presenting cells. Recent advances in single‐cell transcriptomics and lineage‐tracing approaches have revealed that the thymic hematopoietic APC compartment comprises at least seven distinct populations: plasmacytoid DCs, conventional DC1s, conventional DC2s, transitional DCs (tDCs), monocyte‐derived DCs, and two subsets of macrophages (TIM4^+^ resident tissue macrophages and TIM4^−^ monocyte‐derived macrophages) [20, 36, 117]. Each population exhibits unique developmental origins, transcriptional profiles, anatomical distributions, and functional specializations [20, 36].

The thymic microenvironment orchestrates homeostatic APC activation that mimics but is fundamentally different from pathogen‐induced activation in peripheral tissues [72]. This sterile inflammatory environment, characterized by constitutive production of type I and III interferons by mTECs and type 2 cytokines by iNKT cells, drives the maturation of thymic DCs and monocyte‐derived cells into highly activated states [12, 113]. Importantly, this activation occurs independently of pattern recognition receptor signaling and microbiota, representing a tissue‐specific adaptation that expands the repertoire of self‐antigens to include inflammation‐associated and activation‐induced antigens [72, 144].

Thymic DC subsets exhibit a clear division of labor in central tolerance mechanisms. DC1s, particularly their activated CCR7^+^ counterparts, specialize in acquiring and cross‐presenting AIRE‐dependent tissue‐specific antigens from mTECs, primarily driving regulatory T cell inductionp [19, 87]. In contrast, DC2s and their activated subsets appear more involved in clonal deletion of conventional T cells, while also contributing to Treg selection [84, 113]. The newly identified tDCs occupy a unique functional niche by sampling blood‐borne antigens from their perivascular locations, thereby presenting peripheral antigens that may not be captured by other thymic APCs [20].

Fourth, the concept of thymic immigration has been refined to recognize that only specific APC subsets possess the capacity to migrate from peripheral tissues into the thymus. While the majority of thymic DCs develop intrathymically from circulating precursors [79], a subset of DC2s, tDCs, and possibly pDCs can immigrate as mature cells, transporting tissue‐specific antigens from sites such as the intestine and skin [20, 52, 116]. This mechanism ensures that central tolerance extends beyond thymus‐resident antigens to encompass the full spectrum of peripheral self‐antigens.

Despite these advances, several questions remain unresolved and warrant focused investigation. The lack of genetic tools for selective depletion of specific thymic DC subsets remains a major limitation. Future studies should prioritize the development of subset‐specific deletion systems, particularly for DC2s, to definitively establish their individual contributions to T cell selection. The ZEB2 triple‐mutant model, while promising, simultaneously depletes multiple populations and therefore cannot distinguish the specific roles of DC2s versus monocytes and monocyte‐derived cells [20, 97]. Advanced conditional knockout strategies targeting subset‐specific transcription factors or surface markers will be essential.

The mechanisms controlling the magnitude and duration of sterile inflammatory signals in the thymus remain poorly understood. Key questions include: What prevents excessive inflammation that could disrupt normal thymic architecture? How do developmental changes in inflammatory mediators correlate with age‐related thymic involution? Are there feedback mechanisms that adjust inflammatory tone based on thymocyte output or peripheral immune status?

Understanding thymic DC biology opens several therapeutic avenues that merit exploration. These include enhancing thymic DC function to improve immune reconstitution following hematopoietic stem cell transplantation, targeting specific DC subsets to treat autoimmune diseases by promoting tolerance, and modulating thymic inflammation to delay age‐related thymic involution.

In conclusion, while substantial progress has been made in understanding thymic DC biology, the field stands at an exciting juncture where technological advances and conceptual frameworks are converging to enable deeper mechanistic insights. Such efforts will not only advance fundamental immunology but also provide the foundation for novel therapeutic interventions targeting immune tolerance and autoimmunity.

## Conflicts of Interest

The authors declare no conflicts of interest.

## Data Availability

Data sharing not applicable to this article as no datasets were generated or analyzed during the current study.
